# Understanding surface structure and chemistry of single crystal lanthanum aluminate

**DOI:** 10.1038/srep43721

**Published:** 2017-03-02

**Authors:** Stevin S. Pramana, Andrea Cavallaro, Jiahui Qi, Chris L. Nicklin, Mary P. Ryan, Stephen J. Skinner

**Affiliations:** 1 Department of Materials, Imperial College London, Exhibition Road, London, SW7 2AZ, United Kingdom; 2 Diamond Light Source, Harwell Science and Innovation Campus, Didcot, Oxfordshire, OX11 0DE, United Kingdom

## Abstract

The surface crystallography and chemistry of a LaAlO_3_ single crystal, a material mainly used as a substrate to deposit technologically important thin films (e.g. for superconducting and magnetic devices), was analysed using surface X-ray diffraction and low energy ion scattering spectroscopy. The surface was determined to be terminated by Al-O species, and was significantly different from the idealised bulk structure. Termination reversal was not observed at higher temperature (600 °C) and chamber pressure of 10^−10^ Torr, but rather an increased Al-O occupancy occurred, which was accompanied by a larger outwards relaxation of Al from the bulk positions. Changing the oxygen pressure to 10^−6^ Torr enriched the Al site occupancy fraction at the outermost surface from 0.245(10) to 0.325(9). In contrast the LaO, which is located at the next sub-surface atomic layer, showed no chemical enrichment and the structural relaxation was lower than for the top AlO_2_ layer. Knowledge of the surface structure will aid the understanding of how and which type of interface will be formed when LaAlO_3_ is used as a substrate as a function of temperature and pressure, and so lead to improved design of device structures.

Lanthanum aluminate (LaAlO_3_) has been of increasing interest due to its application in the field of two dimensional electron gases (2DEGs) when it is coupled with another insulator. One example is with SrTiO_3_ resulting in superconducting^1^, strong spin-orbit coupling^2^ and magnetic phenomena^3^. This substrate is also used to deposit low frequency dielectric SrTiO_3_^4^ and high critical current density YBa_2_Cu_3_O_7−*x*_ superconducting films^5^. LaAlO_3_ undergoes a structural phase transition from rhombohedral *R*−3*c* to cubic *Pm*-3*m* at ~430–530 °C^6^,^7^,^8^. Whilst the bulk structure of LaAlO_3_ has been documented at different temperatures and pressures, the surface crystallography and chemistry are less well understood due to the lack of characterisation techniques with appropriate sensitivity. However, in order to prepare an excellent interface, crucial for device performance, this knowledge is vital: for example it can directly affect the electronic density of states, valence edge shift and chemical activity. The termination of the LaAlO_3_ (001) surface has been reported to vary with temperature and oxygen partial pressure and there are inconclusive results reported in the literature^9^,^10^,^11^. Using time-of-flight scattering and recoil spectrometry, Yao *et al*.^9^ reported that under an ultra-high vacuum (UHV) environment the surface is terminated by an Al-O layer (from room temperature (RT) to ~150 °C), a mixed Al-O/La-O (150–250 °C) and finally a La-O layer (above 250 °C). In another study using low energy neutral scattering spectroscopy, Kawanowa *et al*.^10^ found at 2 × 10^−10^ Torr a mixed Al-O/La-O (RT) and La-O (727 °C) termination. In addition, a mixed Al-O/La-O termination at 400 °C under 10^−10^ Torr was proposed using data from scanning tunnelling microscopy and ion scattering spectroscopy^11^. Using molecular dynamics simulation, Jacobs *et al*.^12^ suggested that a termination of LaO (1.37 J m^−2^) was energetically favoured over AlO_2_ (1.79 J m^−2^).

Surface X-ray diffraction (SXRD) is now recognised as a very high resolution structural probe of two dimensional crystals in the form of a surface or interface and complementary to other techniques such as scanning tunnelling microscopy and low energy electron diffraction^13^,^14^,^15^. Crystal truncation rod (CTR) profiles^16^,^17^ result from the periodicity perpendicular to the surface plane being broken (‘truncated’), leading to streaks between the Bragg peaks rather than discrete points of intensity^18^,^19^. It is the modulation in intensity along these streaks (resulting from interference between the bulk and surface scattering) that provides high sensitivity to the surface structure and details of the registry with the bulk. CTR measurements are generally carried out using synchrotron radiation (due to the weak surface scattering), and record the intensity as a function of the perpendicular momentum transfer (*ℓ*), for integer values of the in-plane indices (*h* and *k*). The total structure factor^20^,^21^ is given by


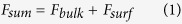


where


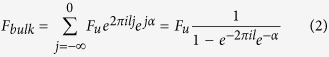



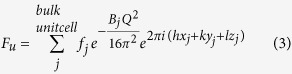



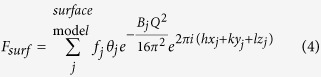


where *f*_*j*_ is the atomic scattering factor of atom *j, B* the Debye-Waller parameter, (*x, y, z*) the atomic coordinate within the unit cell, *α* an attenuation factor and θ the surface occupancy factor.

The only report, by Francis *et al*.^22^, utilising this CTR technique to study the surface structure of LaAlO_3_ described an Al-O termination on the (001) surface at both RT and 400 °C under UHV (1 × 10^−10^ Torr) conditions. At both temperatures, the top surface oxygen position was found to move vertically away from the bulk accompanied by an inward relaxation of the aluminium atom. Their results focused only on measurements of the *h*0*ℓ* rods and specular reflectivity meaning that they were insensitive to the full in-plane relaxation. Instead the lateral movements were constrained using symmetry arguments (and bond distance and valence) to prevent chemically unrealistic solutions.

In this report, we collected a comprehensive set of CTRs, *in-situ* on a single crystal LaAlO_3_ (001) substrate, over a large range of reciprocal space (namely 00*ℓ*, −20*ℓ*, 01*ℓ*, 11*ℓ*, 12*ℓ*, −1−2*ℓ*). The effect of temperature on the surface structure was probed (both below and above the rhombohedral – cubic phase transition (i.e at RT and 600 °C, respectively)) whilst the role of oxygen pressure (10^−10^ and 10^−6^ Torr) was also investigated. This is key information to enable the deposition of high quality La(Ni, Co, Mn)O_3-δ_ and YBa_2_Cu_3_O_7-δ_ thin films for intermediate temperature solid oxide fuel cell cathodes and high temperature superconductors, respectively.

## Results and Discussion

**Figure 1:**
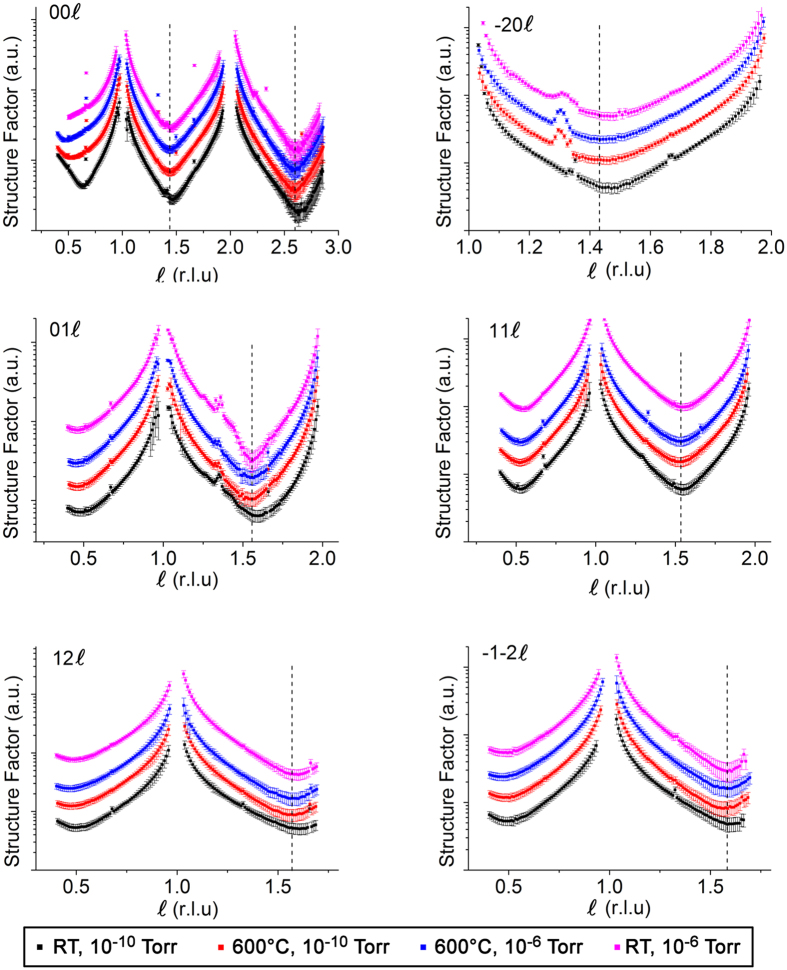
Comparison of 00*ℓ*, −20*ℓ*, 01*ℓ*, 11*ℓ*, 12*ℓ* and −1–2*ℓ* CTR profiles obtained at four different conditions: RT and 10^−10^ Torr (black), 600 °C and 10^−10^ Torr (red), 600 °C and 10^−6^ Torr (blue) and RT and 10^−6^ Torr (magenta). Dashed line is provided as a guide to the eye to indicate the shift in *ℓ*. For −20*ℓ* rod, only a portion of 1.0 ≤ *ℓ* ≤ 2.0 is presented to emphasise the shift in the rod minimum. The small peak observed at ~0 1 1.3 and ~−2 0 1.3 originated from the polycrystalline Ta clip of the sample holder.

**Figure 2:**
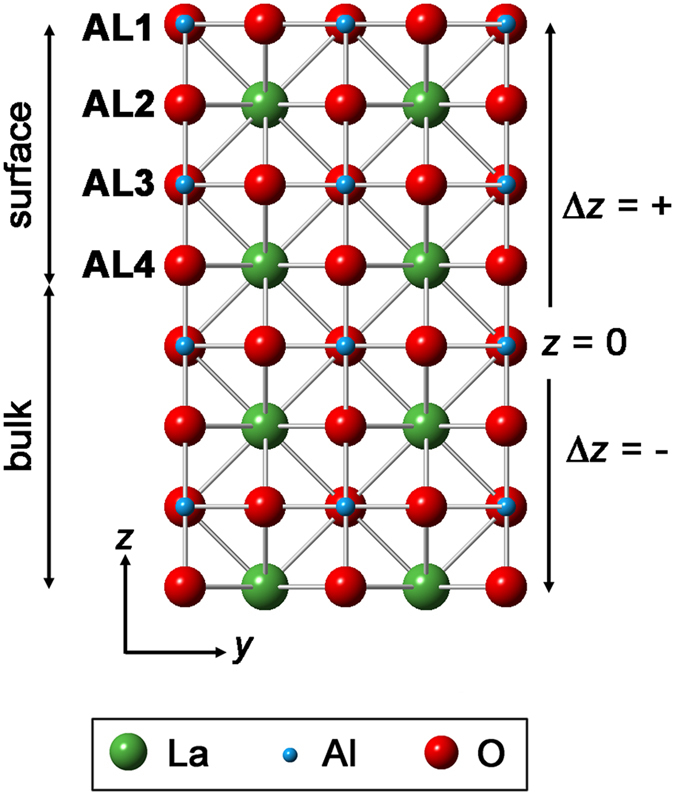
Surface crystal structure representation consisting of four atomic layers (ALs) (AL1: Al(1)O(1a)O(1b), AL2: La(2)O(2), AL3: Al(3)O(3a)O(3b), AL4: La(4)O(4)) that were allowed to relax on top of the ideal bulk structure where the surface starts at *z* = 0. Positive displacement in *z* (Δ*z*) represents the outward displacement towards the vacuum.

**Figure 3:**
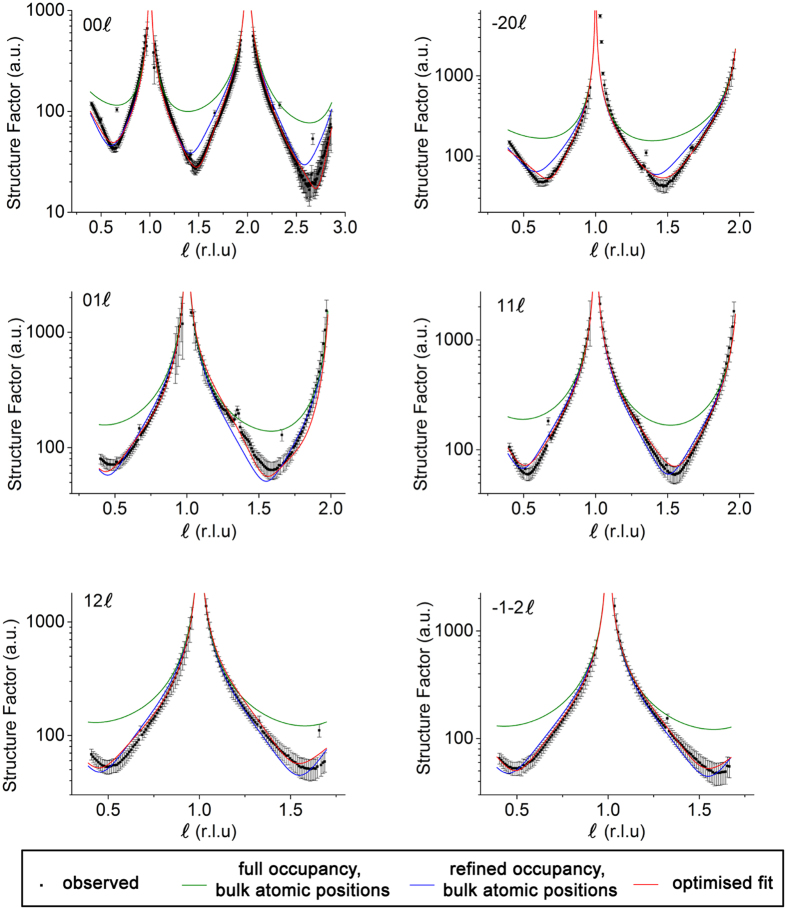
Observed CTRs (dots with error bars) for LaAlO_3_ crystal measured at RT and 10^−10^ Torr and the calculated profiles with full occupancy and bulk atomic positions (green line), refined occupancy and ideal bulk positions (blue line) and the optimised fit with refined atomic positions and occupancy (red line).

Crystal truncation rod (CTR) experiments on the as-received samples demonstrated a very high quality to the surface of the crystal as revealed by the presence of a CTR signal at all positions along the rod (see supplementary information, Fig. S1). It is well known that surface roughness makes the CTR signal disappear in between the Bragg peaks and to reduce the possibility of introducing oxygen vacancies through annealing we chose to use the as-delivered samples as the initial sample condition. The samples were measured under four conditions (i) RT, 10^−10^ Torr, (ii) 600 °C, 10^−10^ Torr, (iii) 600 °C, 10^−6^ Torr of oxygen and (iv) RT, 10^−6^ Torr of oxygen (Fig. S2). A comparison of the CTRs collected at different temperature and chamber pressure conditions is presented in Fig. 1. The first minimum in the rod profiles shifts to lower *ℓ* values for samples measured at higher temperature (600 °C), clearly observed for the −20*ℓ*, 11*ℓ* and 12*ℓ* rods (for example, the minimum shifts from *ℓ* = 1.46 for RT, 10^−10^ Torr to 1.44 for 600 °C, 10^−10^ Torr and 600 °C, 10^−6^ Torr and returns to 1.46 for RT, 10^−6^ Torr for −20*ℓ* rod). However varying the oxygen pressure did not affect the profiles significantly (details of the fits to these data at different conditions are presented in the supplementary information). The small peak observed at ~0 1 1.3 and ~−2 0 1.3 is a powder ring feature, attributed to scattering from the Ta clip of the sample holder and is therefore not included during the fitting. In order to model the surface structure, four atomic layers (LaO – AlO_2_ – LaO – AlO_2_) were included in the surface model (shown in Fig. 2) consistent with the work of Francis *et al*.^22^. A cubic coordinate system to define the crystal lattice was used with a lattice parameter of *a*_1_ = *a*_2_ = *a*_3_ = *a* = 3.7913 Å. The as-received (without any prior cleaning) LaAlO_3_ (001) single crystal substrate measured at RT and 10^−10^ Torr showed a very poor fit (χ^2^ = 10.6) for a simple bulk termination model, which was only marginally improved (χ^2^ = 10.4) when roughness was included as shown in Fig. 3. Whilst non-crystalline surface impurities may remain on the substrate, these would lead only to an increase in the background diffuse scattering and not contribute to the CTR. The surface occupancy of each atomic layer (AlO_2_ and LaO) was the initial fitting parameter refined during the analysis. An occupancy value of 1 corresponds to a complete layer of the bulk terminated structure. The best fits showed the outermost surface to be well oriented and terminated with AlO_2_ but with a reduced occupancy of 0.121(6), whilst the occupancy of the second layer (LaO) was 0.321(2). The next two atomic layers were fully occupied. The atomic positions in the out-of-plane direction (along the sample normal) were subsequently refined, leading to an optimised χ^2^ value of 2.4. Subsequent relaxation of the lateral (in-plane) atomic positions resulted in an improved fit (χ^2^ = 1.9) especially visible for 01 *L* and −1–2*L* CTRs (Fig. S3). The fit to this extensive data set improves on the accuracy of the lateral displacements reported by Francis *et al*.^22^. The best fit parameters are presented in Table 1 and the resulting CTR fit is shown in Fig. 3. The out-of-plane displacements of the Al within the top AlO_2_ layer (Al(1) and Al(3)) were outwards from the bulk positions by 0.10(6) and 0.136(10) Å respectively, accompanied by a smaller inward relaxation of the heavier La(2) and La(4) cations (within the second layer LaO) of −0.090(5) and −0.019(2) Å respectively.

Fits to the data recorded at a higher sample temperature of 600 °C in ultrahigh vacuum indicated an increase in the occupancy of the first atomic layer of AlO_2_ to 0.245(10), whilst that of the second layer LaO reduced slightly to 0.306(2). The data is not consistent with termination reversal to a La-O upper layer at this high temperature, in contrast to reports by Yao *et al*.^9^ and Kawanowa *et al*.^10^ for temperatures above ~250 °C and 727 °C, respectively. The increase in Al occupancy was accompanied by a larger relaxation along the surface normal of Al(1) and Al(3) of 0.26(3) and 0.184(8) Å, respectively (Table 1). The high resolution of the technique is highlighted by the sensitivity to the out-of-plane atomic position of the La(2) atom which has a very strong influence on the position of the first minimum in the CTRs (shifted to lower *ℓ* in the higher temperature data). Specifically we find that Δ*z*_La(2)_ = −0.090(5) Å at RT and −0.032(5) Å at 600 °C leading to detailed information not only about the chemical segregation but also about surface relaxation.

**Figure 4:**
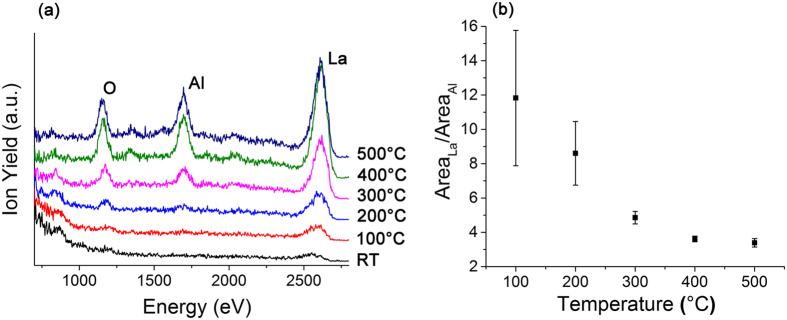
**(a)** Low energy ion scattering spectra of the LaAlO_3_ substrate and **(b)** La/Al area ratio under the peak measured *in-situ* at different temperatures showing a decrease in La/Al as temperature increases. Minor peaks at 846 and 1376 eV are attributed to carbon and fluorine, respectively.

Low energy ion scattering (LEIS) spectroscopy is a highly sensitive technique used to measure the atomic composition of the outermost atomic surface^23^,^24^. At all the temperatures measured, the primary ion yield for La is relatively high. This is consistent with the primary ions being also scattered by the atoms from the second layer LaO layer that are not shielded by the partially occupied outermost AlO_2_ layer. In addition, the scattering cross-section of La is higher than Al^25^. The ratio of the area under the peak of La to Al decreases as the temperature increases, which indicates a surface enrichment of Al at higher temperature (Fig. 4). Minor peaks at 846 and 1376 eV can be attributed to carbon and fluorine contamination, respectively. These species are likely to originate from the heating chamber or during the growth of the single crystal sample. These low atomic number elements have low X-ray scattering factors, and would therefore not contribute significantly to the CTR profiles. Theoretical work by Read *et al*.^26^ and Zhou *et al*.^27^ on La_2_NiO_4_ predicted that the *B* site of *A*_2_*B*O_4_ Ruddlesden-Popper type oxides (*A* = rare earth, alkaline earth; *B* = transition metal) would be the preferential surface termination, in agreement with our data. Experimentally, however, the *B*-O rich surface has not been found in other doped *AB*O_3_ perovskites: La_0.6_Sr_0.4_Co_0.2_Fe_0.8_O_3−δ_ and its derivatives such as the GdBaCo_2_O_5+δ_ double perovskites, and La_2−x_Sr_x_NiO_4+δ_ single crystals characterised by LEIS, CTR and angle-resolved X-ray photoelectron spectroscopy (AR-XPS), were reported to show surface segregation of the *A* cations^28^,^29^. In LaAlO_3_, the absence of transition metals and alkaline earth elements that tend to segregate to the surface^30^,^31^, and are present in other perovskite cathodes may lead to different terminations, which requires further investigation.

Knowledge of the detailed structure of the interface aids in understanding the behaviour of device structures reported in the literature where LaAlO_3_ has been used. For example Liu *et al*.^32^ reported the deposition of SrTiO_3_ films onto LaAlO_3_ substrates at 800 °C in which *n*-type behaviour was expected due to a LaO-TiO_2_ interface, from the presumed La-O termination on the LaAlO_3_ substrate. This *n*-type interface should have shown metallicity characteristic of a two dimensional electron gas (2DEG)^33^. However, insulating behaviour was observed. The nonpolar surface of the LaAlO_3_ substrate (caused by the surface reconstruction), interfacing with nonpolar SrTiO_3_, which supresses the existence of the polar discontinuity, was proposed to be the main reason. However, based on the current study, an AlO_2_ terminated surface would be expected to form a *p*-type interface with SrTiO_3_ which typically requires an extra half a hole or removal of half an electron per surface unit to maintain charge neutrality, by creating an oxygen vacancy (Sr^2+^ O_0.75_^1.5−^)^34^. This *p*-type of interface is reported to be insulating^33^ and may provide an alternative explanation of the observed behaviour.

The surface AlO_2_ occupancy was determined to be even higher (0.325(9)) when oxygen was admitted into the chamber to a pressure of 10^−6^ Torr at 600 °C. This increase in occupancy is only apparent for the outermost species in the first atomic layer whilst the second layer LaO occupancy is relatively stable at 0.3120(17). The top layer Al atoms, Al(1) and Al(3), move further outwards, away from the bulk terminated position compared to the measurements under ultrahigh vacuum. Cooling the single crystal back to RT under the same oxygen pressure (10^−6^ Torr) resulted in a further change to the occupancy of the AlO_2_ outermost surface layer. The occupancy of this layer was refined to be 0.174(9), significantly reduced from that of the surface at 600 °C, but still slightly higher than that determined at the same temperature but under ultrahigh vacuum. The optimised fits under different experimental conditions are shown in Figs S4–S6 whilst fully optimised atomic positions and occupancies are tabulated in Tables S1–S4.

**Figure 5:**
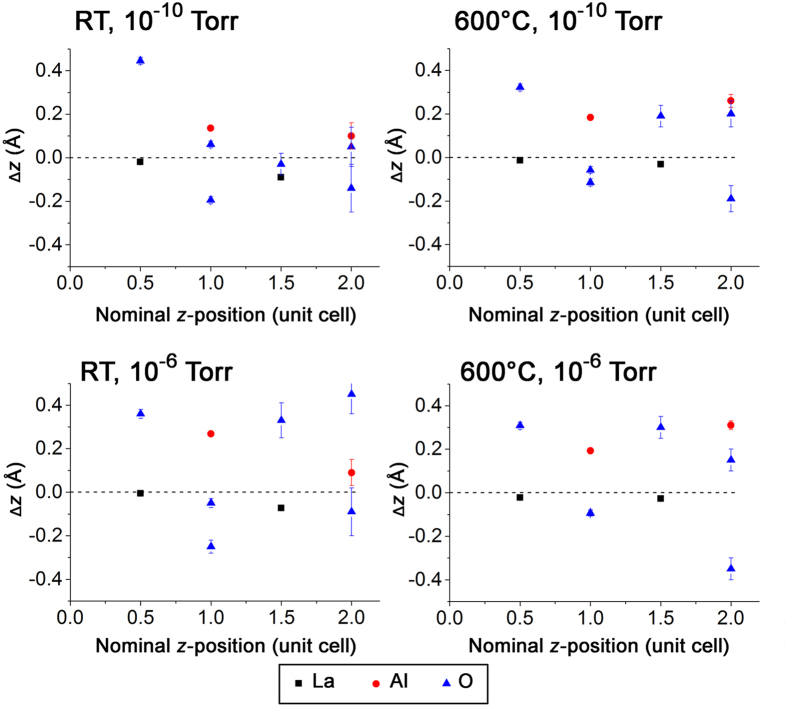
Displacements in *z* (Δ*z*) for La (black square), Al (red circle) and oxygen (blue triangle) at different nominal z-positions. The surface is defined at nominal *z* = 0. Positive and negative Δ*z* refer to a shift towards the vacuum and bulk, respectively. Dashed line indicates that there is no displacement along *z*.

**Figure 6:**
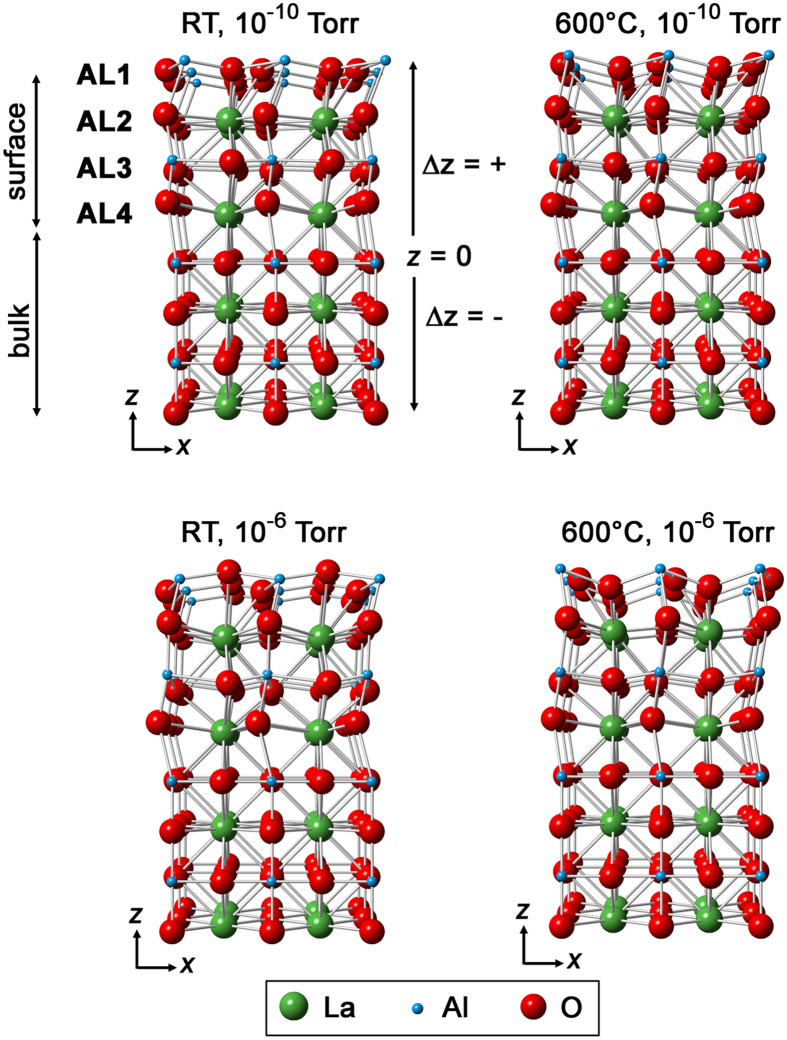
Structural representation projected along [010] comprises the bulk and surface species at different conditions whilst the [100] projection is presented in Fig. S8.

**Figure 7:**
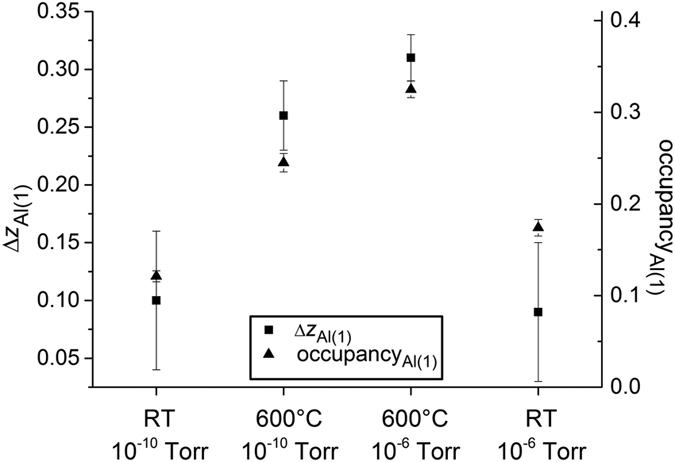
Displacement of *z* (squares) and occupancy of the first atomic layer of Al(1) (triangles) at different experimental conditions showing an increase in Al(1) occupancy as it displaces more towards the vacuum.

The overall displacements of the cations along the surface normal and laterally are presented in Figs 5 and S7 respectively. Structural representations along the [010] and [100] directions are shown in Figs 6 and S8, respectively. The increase in the outer layer occupancy, observed at higher temperature and pressure, is accompanied by an increase in the outward relaxation (Fig. 7). In all experimental conditions, the Al displacement in the outer layer is outwards (away from the bulk) whilst the heavier La displacement in the second layer is always inwards (towards the bulk). A consistent finding was also reported in another non-polar SrTiO_3_ perovskite where Ti was found at the surface with the displacement going away from the bulk for (1 × 1) RT UHV, (2 × 1) RT UHV and (1 × 1) 750 °C, 10^−3^ Pa, except for (2 × 2) RT UHV^35^,^36^.

## Conclusions

In summary, the surface of a LaAlO_3_ single crystal substrate was found to significantly deviate from the ideal bulk structure. The outermost surface is mainly terminated by Al-O species whose occupancy increases with elevated temperature and under higher oxygen pressures, as determined by crystal truncation rod analysis and low energy ion spectroscopy. This is not ideal for in 2DEG applications where an insulating *p*-type interface with SrTiO_3_ is likely to form. The La-O in the second atomic layer is not fully occupied but was relatively unmodified over the range of temperatures and pressures studied. Under all conditions there was an outwards relaxation of the AlO_2_ terminating layer, which increased at higher temperatures and pressures, whilst the second layer La cations showed a smaller inward relaxation (≤0.090(5) Å) that was independent of the measurement conditions.

## Methods

### Crystal Truncation Rods

Single crystal LaAlO_3_ (001) single-side polished substrates (5 × 5 mm^2^) were obtained from CrysTec Kristalltechnologie. The CTR measurements were carried out at the ultrahigh vacuum endstation of beamline I07^37^ of Diamond Light Source, using an X-ray energy of 12.2 keV (λ = 1.01627 Å), a hexapod sample mount for surface alignment and a two-dimensional Pilatus 100 K detector. The specular reflectivity (00*ℓ*) was recorded using a θ–2θ geometry (incidence angle = exit angle) with the surface normal horizontal while the CTRs were recorded with a fixed incidence angle of 0.5°. The structure factor was calculated from each image by taking the background subtracted integrated intensity of the CTR and applying correction factors to account for the polarisation correction and rod interception, prior to taking the square root. Data fitting was performed using the ROD and WinRod programs^20^.

### Low Energy Ion Scattering

One of the as-received LaAlO_3_ (001) samples was also analysed using low energy ion scattering (LEIS, Qtac100 spectrometer, IonTOF Gmbh, Germany). The primary beam was a 3 keV ^4^He^+^, positioned normal to the surface with a collection angle of 145°. The sampled raster area of the LaAlO_3_ substrate used was 750 × 750 μm^2^. The spectra were measured *in-situ* at different temperatures and a chamber pressure of 10^−9^ Torr using an ion current of ~5 nA. The spectra were fitted using SurfaceLab 6 software (IonTOF Gmbh, Germany) with Gaussian peaks of different elemental components with the background described by error functions^38^,^39^.

## Additional Information

**How to cite this article:** Pramana, S. S. *et al*. Understanding surface structure and chemistry of single crystal lanthanum aluminate. *Sci. Rep.* **7**, 43721; doi: 10.1038/srep43721 (2017).

**Publisher's note:** Springer Nature remains neutral with regard to jurisdictional claims in published maps and institutional affiliations.

## Supplementary Material

Supplementary Information

## Figures and Tables

**Table 1:** Bulk-terminated atomic positions and optimised displacements (Δ) and occupancy of the surface (four atomic layers) at different conditions.

Atomic Layer (AL)		bulk-terminated			optimised		condition
		*x*	*y*	*z*	Δ*z*(Å)	occupancy	
1	Al(1)	0	0	2	0.10 (6)	0.121 (6)	RT, 10^−10^ Torr
					0.26 (3)	0.245 (10)	600 °C, 10^−10^ Torr
					0.31 (2)	0.325 (9)	600 °C, 10^−6^ Torr
					0.09 (6)	0.174 (9)	RT, 10^−6^ Torr
	O(1a)	0.5	0	2	−0.14 (11)	0.121 (6)	RT, 10^−10^ Torr
					−0.19 (6)	0.245 (10)	600 °C, 10^−10^ Torr
					−0.35 (5)	0.325 (9)	600 °C, 10^−6^ Torr
					0.45 (9)	0.174 (9)	RT, 10^−6^ Torr
	O(1b)	0	0.5	2	0.05 (9)	0.121 (6)	RT, 10^−10^ Torr
					0.20 (6)	0.245 (10)	600 °C, 10^−10^ Torr
					0.15 (5)	0.325 (9)	600 °C, 10^−6^ Torr
					−0.09 (11)	0.174 (9)	RT, 10^−6^ Torr
2	La(2)	0.5	0.5	1.5	−0.090 (5)	0.321 (2)	RT, 10^−10^ Torr
					−0.032 (5)	0.306 (2)	600 °C, 10^−10^ Torr
					−0.027 (5)	0.3120 (17)	600 °C, 10^−6^ Torr
					−0.073 (6)	0.310 (3)	RT, 10^−6^ Torr
	O(2)	0	0	1.5	−0.03 (5)	0.321 (2)	RT, 10^−10^ Torr
					0.19 (5)	0.306 (2)	600 °C, 10^−10^ Torr
					0.30 (5)	0.3120 (17)	600 °C, 10^−6^ Torr
					0.33 (8)	0.310 (3)	RT, 10^−6^ Torr
3	Al(3)	0	0	1	0.136 (10)	1	RT, 10^−10^ Torr
					0.184 (8)	1	600 °C, 10^−10^ Torr
					0.193 (8)	1	600 °C, 10^−6^ Torr
					0.268 (12)	1	RT, 10^−6^ Torr
	O(3a)	0.5	0	1	0.061 (16)	1	RT, 10^−10^ Torr
					−0.058 (16)	1	600 °C, 10^−10^ Torr
					−0.094 (14)	1	600 °C, 10^−6^ Torr
					−0.05 (2)	1	RT, 10^−6^ Torr
	O(3b)	0	0.5	1	−0.195 (16)	1	RT, 10^−10^ Torr
					−0.115 (16)	1	600 °C, 10^−10^ Torr
					−0.093 (14)	1	600 °C, 10^−6^ Torr
					−0.25 (3)	1	RT, 10^−6^ Torr
4	La(4)	0.5	0.5	0.5	−0.019 (2)	1	RT, 10^−10^ Torr
					−0.014 (3)	1	600 °C, 10^−10^ Torr
					−0.022 (3)	1	600 °C, 10^−6^ Torr
					−0.006 (4)	1	RT, 10^−6^ Torr
	O(4)	0	0	0.5	0.445 (16)	1	RT, 10^−10^ Torr
					0.322 (16)	1	600 °C, 10^−10^ Torr
					0.308 (16)	1	600 °C, 10^−6^ Torr
					0.36 (2)	1	RT, 10^−6^ Torr

The occupancy was constrained to have an identical value for Al(1)O(1a)O(1b) and La(2)O(2). Bulk unit cell adopted Al (0, 0, 0), La (0.5, 0.5, −0.5) and O((0.5, 0, 0), (0, 0.5, 0), (0, 0, −0.5)).
