# Supplementary material for: Understanding surface structure and chemistry of single crystal lanthanum aluminate
Source: Sci Rep. 2017 Mar 2;7:43721. doi: 10.1038/srep43721 (PMC5333088; doi:10.1038/srep43721)
Supplement: Supplementary Information [file srep43721-s1.pdf]

**Supplementary Information**

# Understanding surface structure and chemistry of single crystal lanthanum aluminate

Stevin S. Pramana,[1] Andrea Cavallaro,[1] Jiahui Qi,[1] Chris L. Nicklin,[2] Mary P. Ryan,[1]

Stephen J. Skinner[1]

[1]Department of Materials, Imperial College London, Exhibition Road, London, SW7 2AZ, United Kingdom.

[2]Diamond Light Source, Harwell Science and Innovation Campus, Didcot, Oxfordshire, OX11 0DE, United Kingdom.

Correspondence and requests for materials should be addressed to S.S.P (email: s.pramana@imperial.ac.uk) and S.J.S (email: s.skinner@imperial.ac.uk)

Keywords: surface science; crystal truncation rod; crystallography; lanthanum aluminate; low energy ion scattering; single crystal

**Table S1.** Bulk-terminated and optimised atomic positions and occupancy of the surface model at RT, $10^{-10}$ Torr with the displacement (Δ) of the latter positions with respect to the former. The occupancy was constrained to have the identical value for Al(1)O(1a)O(1b) and La(2)O(2).

| **Atomic Layer (AL)** | | **Bulk-terminated** | | | **Optimised** | | | | | | |
|---|---|---|---|---|---|---|---|---|---|---|---|
| | | ***x*** | ***y*** | ***z*** | ***x*** | ***y*** | ***z*** | **occupancy** | **Δ*x* (Å)** | **Δ*y* (Å)** | **Δ*z* (Å)** |
| 1 | Al(1) | 0 | 0 | 2 | 0.09(2) | -0.08(3) | 2.026(15) | 0.121(6) | 0.35(8) | -0.29(12) | 0.10(6) |
| | O(1a) | 0.5 | 0 | 2 | 0.56(3) | 0.14(3) | 1.96(3) | 0.121(6) | 0.23(12) | 0.51(12) | -0.14(11) |
| | O(1b) | 0 | 0.5 | 2 | -0.15(3) | 0.63(3) | 2.01(3) | 0.121(6) | -0.55(12) | 0.48(12) | 0.05(9) |
| 2 | La(2) | 0.5 | 0.5 | 1.5 | 0.5141(15) | 0.492(2) | 1.4763(13) | 0.321(2) | 0.054(6) | -0.030(8) | -0.090(5) |
| | O(2) | 0 | 0 | 1.5 | -0.103(17) | -0.004(17) | 1.493(12) | 0.321(2) | -0.39(6) | -0.02(6) | -0.03(5) |
| 3 | Al(3) | 0 | 0 | 1 | -0.034(3) | -0.017(3) | 1.036(3) | 1 | -0.130(10) | -0.063(12) | 0.136(10) |
| | O(3a) | 0.5 | 0 | 1 | 0.562(6) | -0.070(6) | 1.016(4) | 1 | 0.24(2) | -0.26(2) | 0.061(16) |
| | O(3b) | 0 | 0.5 | 1 | -0.045(6) | 0.533(6) | 0.949(4) | 1 | -0.17(2) | 0.12(2) | -0.195(16) |
| 4 | La(4) | 0.5 | 0.5 | 0.5 | 0.4993(6) | 0.4989(16) | 0.4950(6) | 1 | -0.003(2) | -0.004(6) | -0.019(2) |
| | O(4) | 0 | 0 | 0.5 | -0.086(8) | 0.039(6) | 0.617(4) | 1 | -0.33(3) | 0.15(2) | 0.445(16) |
| | $Al_{bulk}$ | 0 | 0 | 0 | 0 | 0 | 0 | 1 | 0 | 0 | 0 |
| | $O_{bulk}$ | 0.5 | 0 | 0 | 0.5 | 0 | 0 | 1 | 0 | 0 | 0 |
| | $O_{bulk}$ | 0 | 0.5 | 0 | 0 | 0.5 | 0 | 1 | 0 | 0 | 0 |
| | $La_{bulk}$ | 0.5 | 0.5 | -0.5 | 0.5 | 0.5 | -0.5 | 1 | 0 | 0 | 0 |
| | $O_{bulk}$ | 0 | 0 | -0.5 | 0 | 0 | -0.5 | 1 | 0 | 0 | 0 |

**Table S2.** Bulk-terminated and optimised atomic positions and occupancy of the surface model at 600°C, $10^{-10}$ Torr with the displacement (Δ) of the latter positions with respect to the former. The occupancy was constrained to have the identical value for Al(1)O(1a)O(1b) and La(2)O(2).

| **Atomic Layer (AL)** | | **Bulk-terminated** | | | **Optimised** | | | | | | |
|---|---|---|---|---|---|---|---|---|---|---|---|
| | | ***x*** | ***y*** | ***z*** | ***x*** | ***y*** | ***z*** | **occupancy** | **Δ*x* (Å)** | **Δ*y* (Å)** | **Δ*z* (Å)** |
| 1 | Al(1) | 0 | 0 | 2 | 0.062(11) | -0.068(11) | 2.068(9) | 0.245(10) | 0.23(5) | -0.26(5) | 0.26(3) |
| | O(1a) | 0.5 | 0 | 2 | 0.571(17) | 0.03(2) | 1.950(15) | 0.245(10) | 0.27(6) | 0.13(8) | -0.19(6) |
| | O(1b) | 0 | 0.5 | 2 | -0.04(2) | 0.53(2) | 2.05(2) | 0.245(10) | -0.17(8) | 0.11(9) | 0.20(6) |
| 2 | La(2) | 0.5 | 0.5 | 1.5 | 0.5095(15) | 0.495(2) | 1.4917(15) | 0.306(2) | 0.036(6) | -0.019(8) | -0.032(5) |
| | O(2) | 0 | 0 | 1.5 | -0.057(15) | -0.036(15) | 1.549(12) | 0.306(2) | -0.21(6) | -0.14(6) | 0.19(5) |
| 3 | Al(3) | 0 | 0 | 1 | -0.014(3) | -0.007(3) | 1.049(2) | 1 | -0.054(10) | -0.025(12) | 0.184(8) |
| | O(3a) | 0.5 | 0 | 1 | 0.523(6) | 0.022(6) | 0.985(4) | 1 | 0.09(2) | 0.08(2) | -0.058(16) |
| | O(3b) | 0 | 0.5 | 1 | -0.058(4) | 0.573(6) | 0.970(4) | 1 | -0.221(16) | 0.28(2) | -0.115(16) |
| 4 | La(4) | 0.5 | 0.5 | 0.5 | 0.4977(6) | 0.5082(14) | 0.4963(8) | 1 | -0.009(2) | 0.031(6) | -0.014(3) |
| | O(4) | 0 | 0 | 0.5 | -0.106(8) | 0.071(6) | 0.585(4) | 1 | -0.40(3) | 0.27(2) | 0.322(16) |
| | $Al_{bulk}$ | 0 | 0 | 0 | 0 | 0 | 0 | 1 | 0 | 0 | 0 |
| | $O_{bulk}$ | 0.5 | 0 | 0 | 0.5 | 0 | 0 | 1 | 0 | 0 | 0 |
| | $O_{bulk}$ | 0 | 0.5 | 0 | 0 | 0.5 | 0 | 1 | 0 | 0 | 0 |
| | $La_{bulk}$ | 0.5 | 0.5 | -0.5 | 0.5 | 0.5 | -0.5 | 1 | 0 | 0 | 0 |
| | $O_{bulk}$ | 0 | 0 | -0.5 | 0 | 0 | -0.5 | 1 | 0 | 0 | 0 |

**Table S3.** Bulk-terminated and optimised atomic positions and occupancy of the surface model at 600°C, $10^{-6}$ Torr with the displacement (Δ) of the latter positions with respect to the former. The occupancy was constrained to have the identical value for Al(1)O(1a)O(1b) and La(2)O(2).

| **Atomic Layer (AL)** | | **Bulk-terminated** | | | **Optimised** | | | | | | |
|---|---|---|---|---|---|---|---|---|---|---|---|
| | | ***x*** | ***y*** | ***z*** | ***x*** | ***y*** | ***z*** | **occupancy** | **Δ*x* (Å)** | **Δ*y* (Å)** | **Δ*z* (Å)** |
| 1 | Al(1) | 0 | 0 | 2 | -0.010(8) | -0.030(8) | 2.081(6) | 0.325(9) | -0.04(3) | -0.11(3) | 0.31(2) |
| | O(1a) | 0.5 | 0 | 2 | 0.485(15) | 0.042(15) | 1.908(11) | 0.325(9) | -0.06(5) | 0.16(5) | -0.35(5) |
| | O(1b) | 0 | 0.5 | 2 | 0.147(12) | 0.473(15) | 2.040(11) | 0.325(9) | 0.56(5) | -0.10(6) | 0.15(5) |
| 2 | La(2) | 0.5 | 0.5 | 1.5 | 0.5003(15) | 0.4945(15) | 1.4930(12) | 0.3120(17) | 0.001(5) | -0.021(6) | -0.027(5) |
| | O(2) | 0 | 0 | 1.5 | 0.063(12) | -0.098(15) | 1.579(11) | 0.3120(17) | 0.24(5) | -0.37(5) | 0.30(5) |
| 3 | Al(3) | 0 | 0 | 1 | -0.014(3) | 0.007(3) | 1.0509(2) | 1 | -0.052(10) | 0.025(10) | 0.193(8) |
| | O(3a) | 0.5 | 0 | 1 | 0.514(6) | 0.053(6) | 0.975(4) | 1 | 0.05(2) | 0.20(2) | -0.094(14) |
| | O(3b) | 0 | 0.5 | 1 | -0.056(4) | 0.586(6) | 0.976(4) | 1 | -0.210(16) | 0.33(2) | -0.093(14) |
| 4 | La(4) | 0.5 | 0.5 | 0.5 | 0.4973(6) | 0.5092(12) | 0.4943(8) | 1 | -0.010(2) | 0.035(4) | -0.022(3) |
| | O(4) | 0 | 0 | 0.5 | -0.091(6) | 0.070(6) | 0.581(4) | 1 | -0.34(2) | 0.26(2) | 0.308(16) |
| | $Al_{bulk}$ | 0 | 0 | 0 | 0 | 0 | 0 | 1 | 0 | 0 | 0 |
| | $O_{bulk}$ | 0.5 | 0 | 0 | 0.5 | 0 | 0 | 1 | 0 | 0 | 0 |
| | $O_{bulk}$ | 0 | 0.5 | 0 | 0 | 0.5 | 0 | 1 | 0 | 0 | 0 |
| | $La_{bulk}$ | 0.5 | 0.5 | -0.5 | 0.5 | 0.5 | -0.5 | 1 | 0 | 0 | 0 |
| | $O_{bulk}$ | 0 | 0 | -0.5 | 0 | 0 | -0.5 | 1 | 0 | 0 | 0 |

**Table S4.** Bulk-terminated and optimised atomic positions and occupancy of the surface model at RT, $10^{-6}$ Torr with the displacement (Δ) of the latter positions with respect to the former. The occupancy was constrained to have the identical value for Al(1)O(1a)O(1b) and La(2)O(2).

| Atomic Layer (AL) | | Bulk-terminated | | | Optimised | | | | | | |
|---|---|---|---|---|---|---|---|---|---|---|---|
| | | ***x*** | ***y*** | ***z*** | ***x*** | ***y*** | ***z*** | **occupancy** | **Δ*x* (Å)** | **Δ*y* (Å)** | **Δ*z* (Å)** |
| 1 | Al(1) | 0 | 0 | 2 | 0.076(17) | -0.01(2) | 2.025(15) | 0.174(9) | 0.29(6) | -0.05(9) | 0.09(6) |
| | O(1a) | 0.5 | 0 | 2 | 0.56(5) | 0.14(5) | 2.12(3) | 0.174(9) | 0.24(17) | 0.52(17) | 0.45(9) |
| | O(1b) | 0 | 0.5 | 2 | -0.10(3) | 0.63(5) | 1.98(3) | 0.174(9) | -0.38(12) | 0.47(17) | -0.09(11) |
| 2 | La(2) | 0.5 | 0.5 | 1.5 | 0.515(2) | 0.489(2) | 1.481(2) | 0.310(3) | 0.058(6) | -0.044(9) | -0.073(6) |
| | O(2) | 0 | 0 | 1.5 | -0.03(2) | -0.05(2) | 1.59(2) | 0.310(3) | -0.10(9) | -0.19(8) | 0.33(8) |
| 3 | Al(3) | 0 | 0 | 1 | -0.045(3) | -0.005(4) | 1.071(3) | 1 | -0.170(12) | -0.019(14) | 0.268(12) |
| | O(3a) | 0.5 | 0 | 1 | 0.564(8) | -0.056(6) | 0.987(6) | 1 | 0.24(3) | -0.21(2) | -0.05(2) |
| | O(3b) | 0 | 0.5 | 1 | -0.002(10) | 0.543(8) | 0.934(8) | 1 | -0.01(4) | 0.16(3) | -0.25(3) |
| 4 | La(4) | 0.5 | 0.5 | 0.5 | 0.4994(8) | 0.4954(18) | 0.4985(10) | 1 | -0.002(4) | -0.017(6) | -0.006(4) |
| | O(4) | 0 | 0 | 0.5 | -0.143(8) | 0.086(8) | 0.595(6) | 1 | -0.54(3) | 0.33(3) | 0.36(2) |
| | $Al_{bulk}$ | 0 | 0 | 0 | 0 | 0 | 0 | 1 | 0 | 0 | 0 |
| | $O_{bulk}$ | 0.5 | 0 | 0 | 0.5 | 0 | 0 | 1 | 0 | 0 | 0 |
| | $O_{bulk}$ | 0 | 0.5 | 0 | 0 | 0.5 | 0 | 1 | 0 | 0 | 0 |
| | $La_{bulk}$ | 0.5 | 0.5 | -0.5 | 0.5 | 0.5 | -0.5 | 1 | 0 | 0 | 0 |
| | $O_{bulk}$ | 0 | 0 | -0.5 | 0 | 0 | -0.5 | 1 | 0 | 0 | 0 |

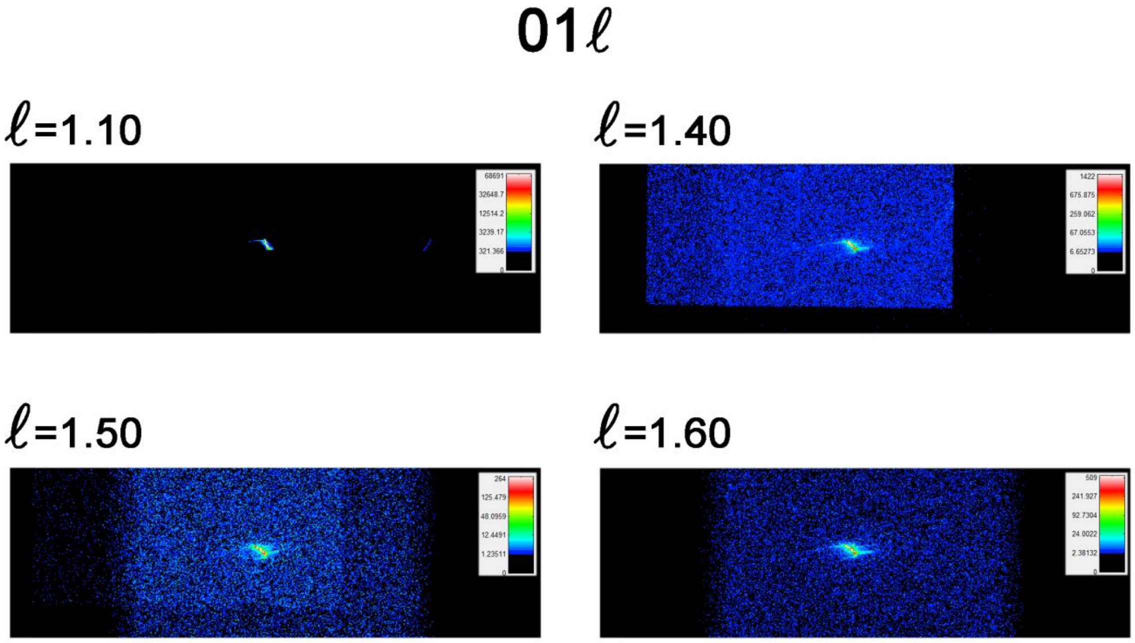


**Figure S1.** Two-dimensional SXRD data obtained with a Pilatus detector showing that the CTR is relatively sharp at anti-Bragg peaks, indicating a smooth surface.

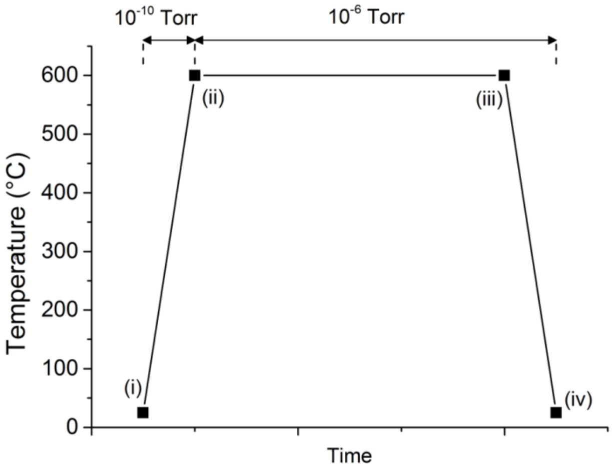


**Figure S2.** Crystal truncation rod experimental conditions.

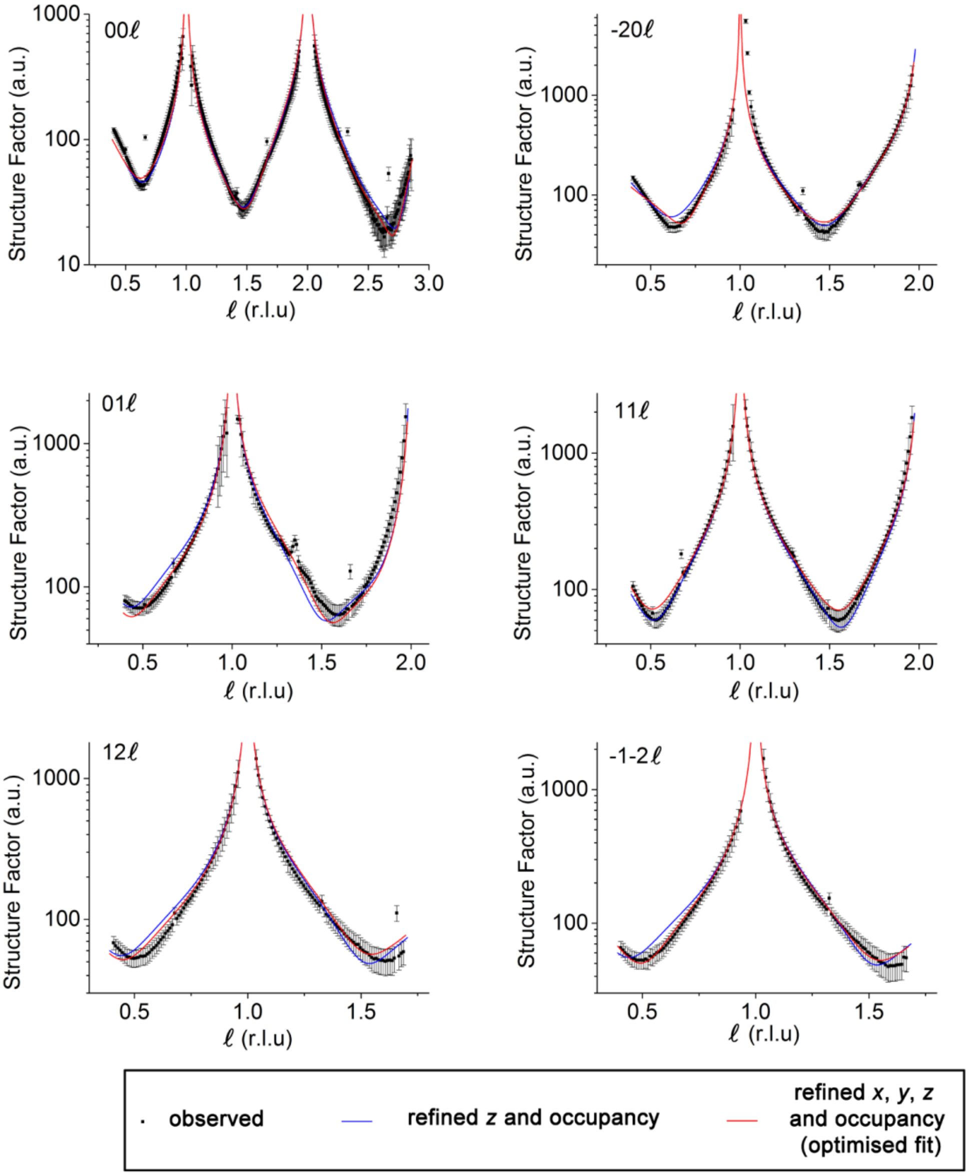


**Figure S3.** Observed (dots with error bars) CTRs and optimised fit, with refined fractional coordinates *x, y, z*, and occupancy, calculated profiles (red line) for $LaAlO_3$ crystal at RT and $10^{-10}$ Torr. Blue line represents the best fit with refinement of *only* occupancy and fractional coordinate *z*. The comparison of the two fits highlights the difference of *only* out-of-plane fractional coordinate *z* relaxation combined with refined occupancy, and in-plane/lateral (*x* and *y*) and out-of-plane (*z*) relaxation with refined occupancy.

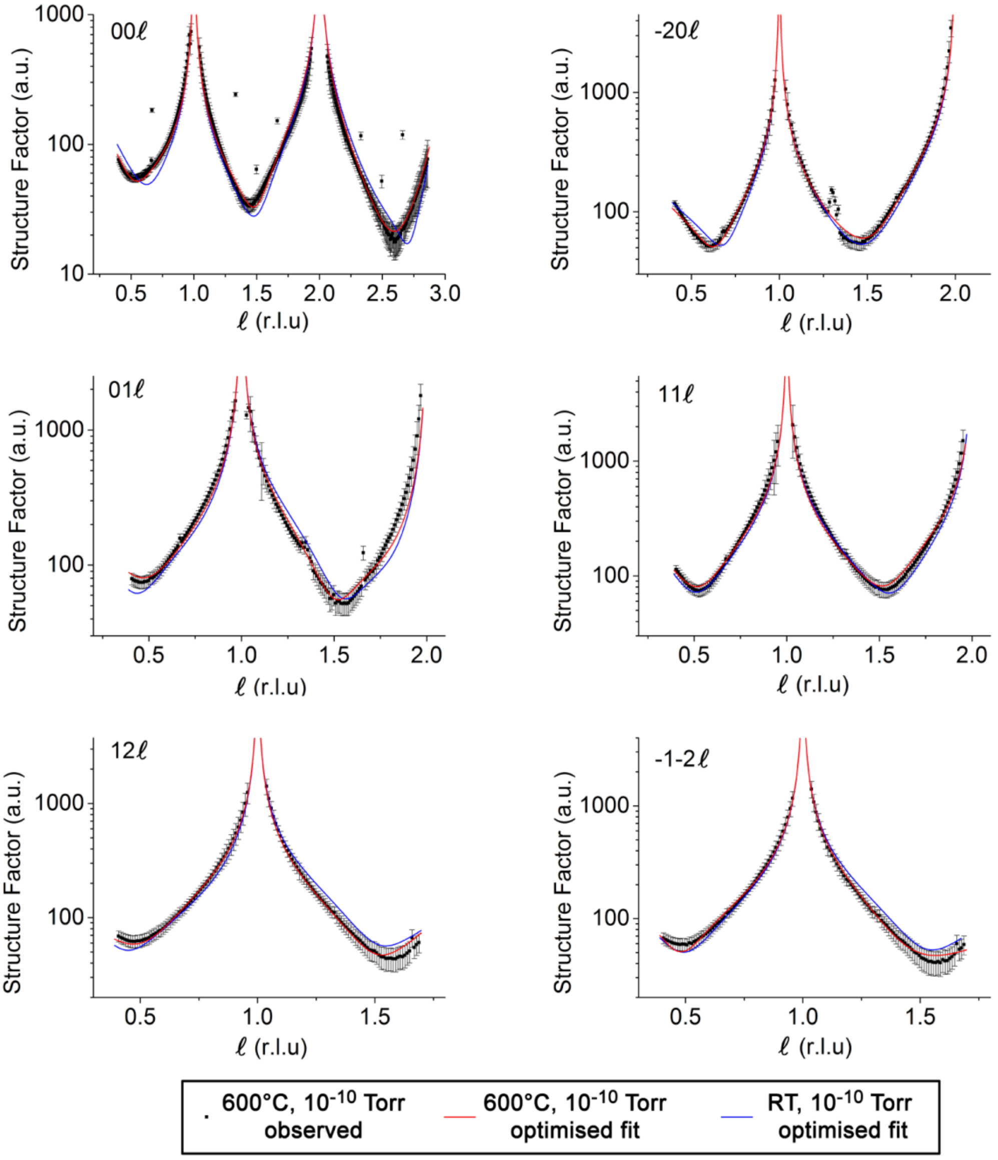


**Figure S4.** Observed (dots with error bars) CTRs and optimised fit profiles (red line) for $LaAlO_3$ crystal at 600°C and $10^{-10}$ Torr. In order to emphasise the effect of temperature variation, the optimised fit at RT and $10^{-10}$ Torr is plotted as a blue line, for comparison.

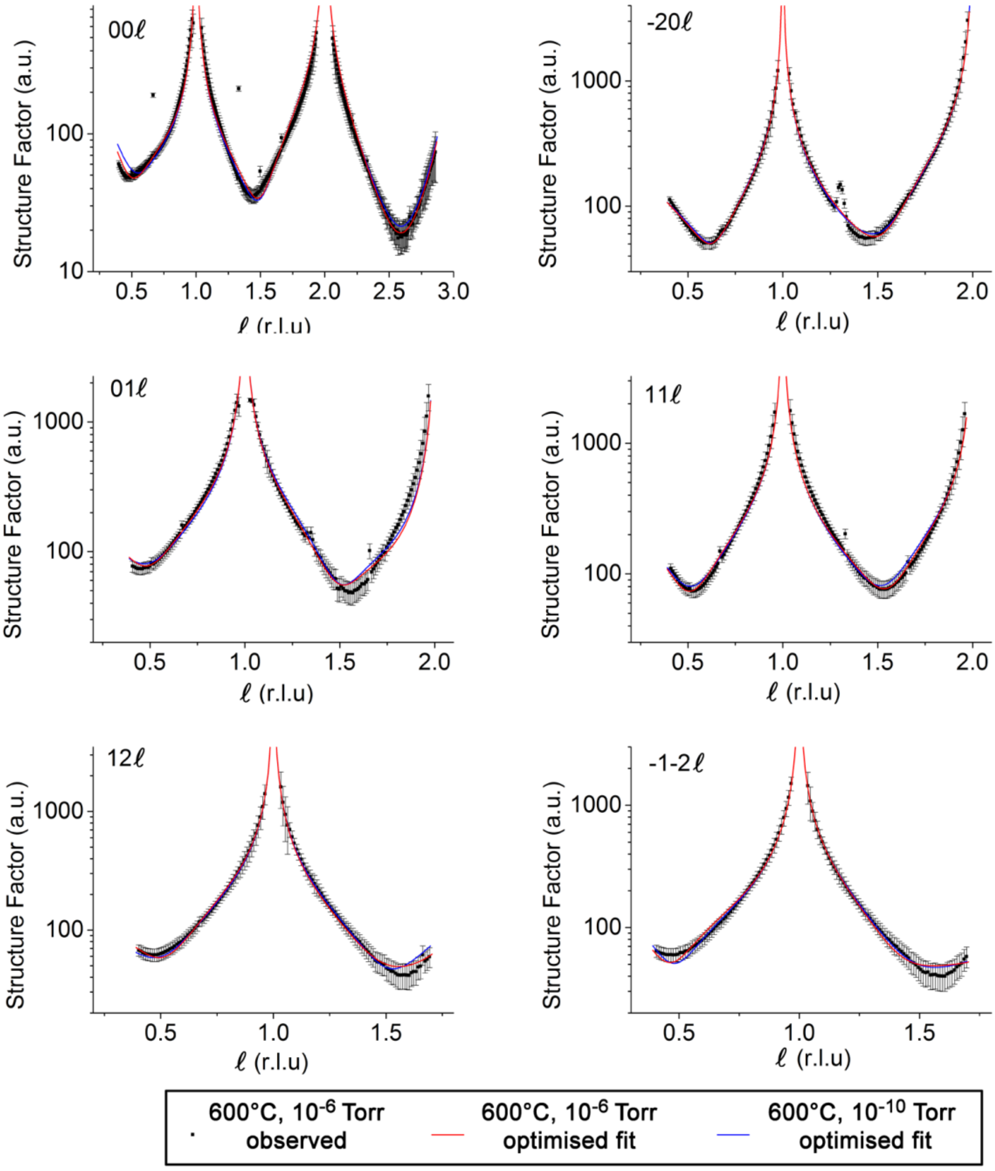


**Figure S5.** Observed (dots with error bars) CTRs and optimised fit calculated profiles (red line) for $LaAlO_3$ crystal at 600°C and $10^{-6}$ Torr. In order to emphasise the effect of pressure variation the optimised fit at 600°C and $10^{-10}$ Torr is plotted as a blue line, for comparison.

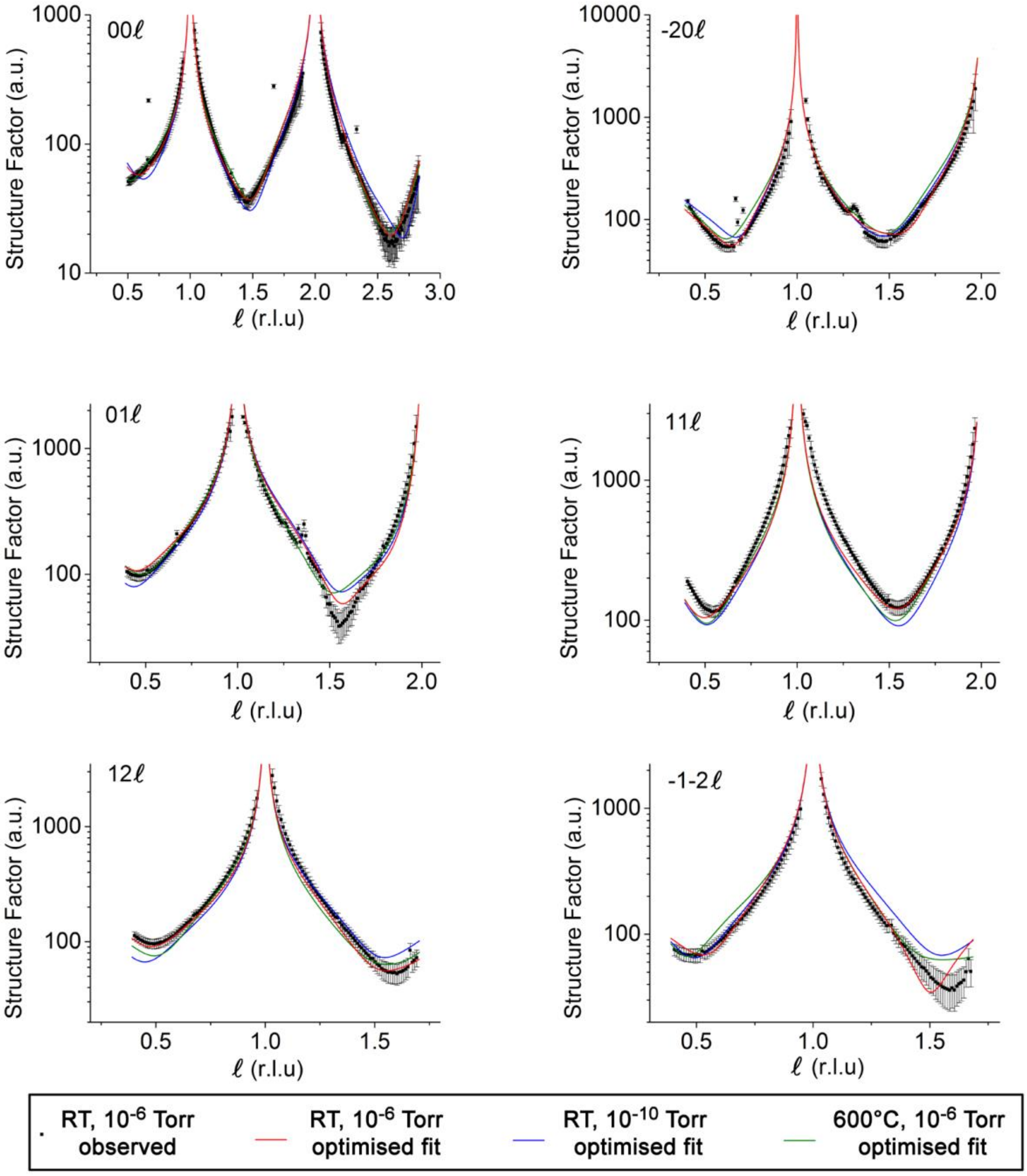


**Figure S6.** Observed (dots with error bars) CTRs and optimised fit calculated profiles (red line) for $LaAlO_3$ crystal at RT and $10^{-6}$ Torr. Blue and green lines represent the best fit at RT, $10^{-10}$ Torr and 600°C, $10^{-6}$ Torr, respectively, for comparison.

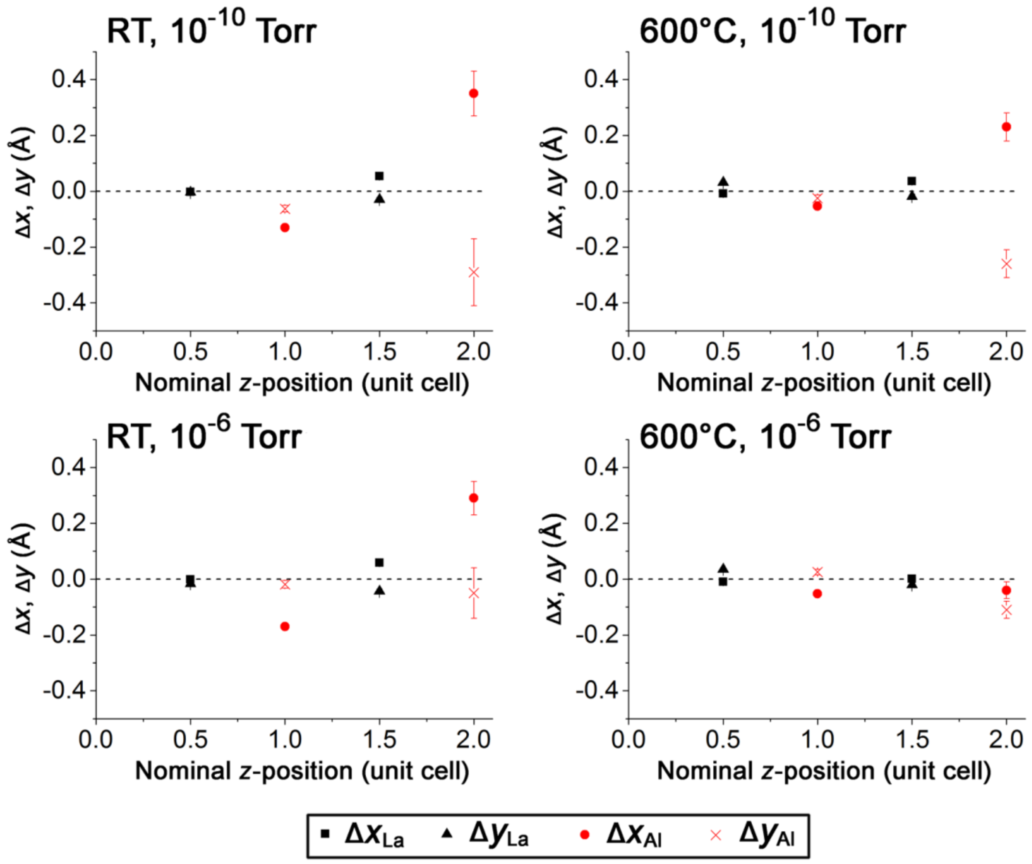


**Figure S7.** La and Al displacements in *x* (black square: $\Delta x_{La}$, red circle: $\Delta x_{Al}$) and *y* (black triangle: $\Delta y_{La}$, red cross: $\Delta y_{Al}$) at different nominal *z*-positions. Dashed line indicates that there is no displacement along *x* or *y*.

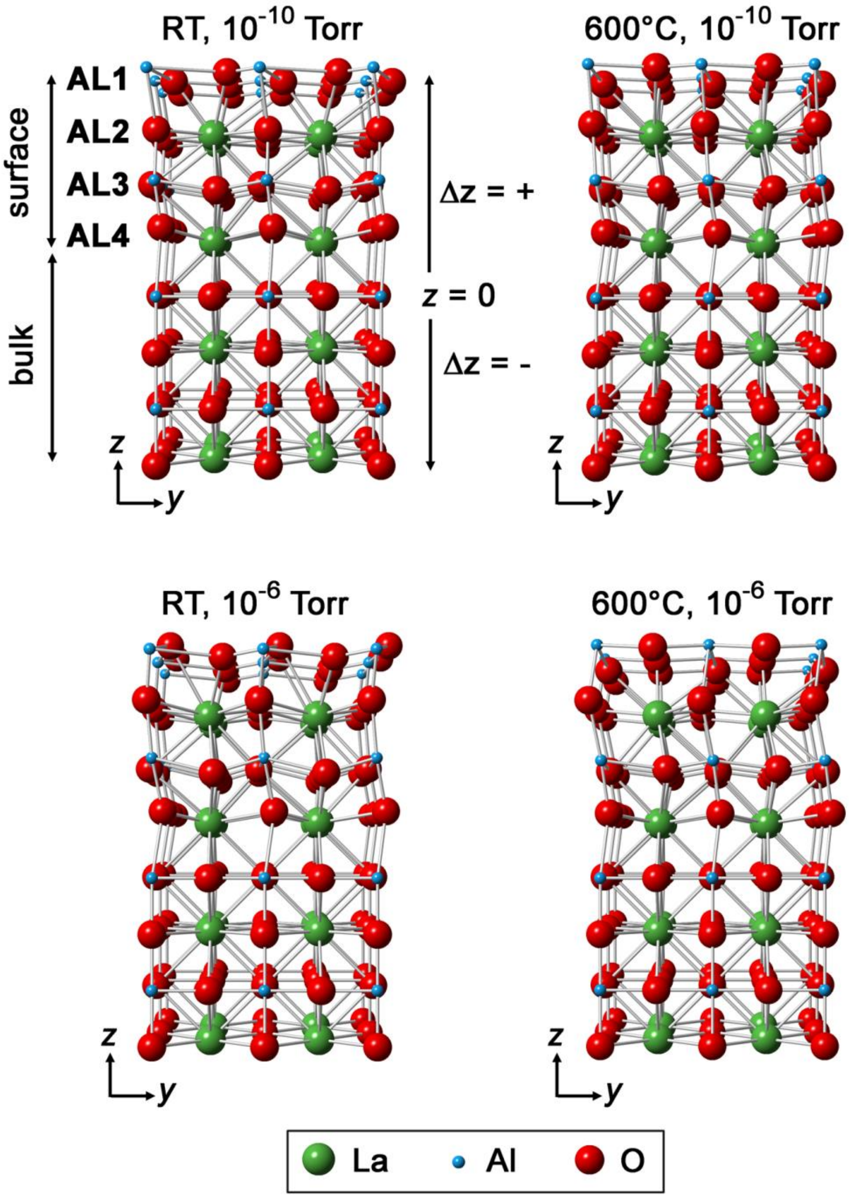


**Figure S8.** Structural representation projected along [100] comprises the bulk and surface species at different temperature and pressure conditions.
